# Heat stress induces calcium dyshomeostasis to subsequent cognitive impairment through ERS-mediated apoptosis via SERCA/PERK/eIF2α pathway

**DOI:** 10.1038/s41420-024-02047-7

**Published:** 2024-06-11

**Authors:** Hongxia Li, Wenlan Pan, Chenqi Li, Mengyu Cai, Wenjing Shi, Zifu Ren, Hongtao Lu, Qicheng Zhou, Hui Shen

**Affiliations:** 1 Department of Nutrition and Food Hygiene, Faculty of Naval Medicine, Naval Medical University, Shanghai, 200433 China; 2 Department of Clinical Nutrition, Shanghai Ninth People’s Hospital, Shanghai JiaoTong University School of Medicine, Shanghai, 201999 China; 3 Department of Nutrition, The Third Affiliated Hospital of Naval Medical University, Shanghai, 200438 China

**Keywords:** Disorders of consciousness, Hippocampus

## Abstract

Heat exposure is an environmental stressor that has been associated with cognitive impairment. However, the neural mechanisms that underlie this phenomenon have yet to be extensively investigated. The Morris water maze test was utilized to assess cognitive performance. RNA sequencing was employed to discover the primary regulators and pathological pathways involved in cognitive impairment caused by heat. Before heat exposure in vivo and in vitro, activation of the sarco/endoplasmic reticulum (SR/ER) calcium (Ca^2+^)-ATPase (SERCA) was achieved by CDN1163. Hematoxylin-Eosin, Nissl staining, calcium imaging, transmission electron microscopy, western blot, and immunofluorescence were utilized to visualize histological changes, intracellular calcium levels, endoplasmic reticulum stress (ERS) markers, apoptosis, and synaptic proteins alterations. Heat stress (HS) significantly induced cognitive decline and neuronal damage in mice. By the transcriptome sequencing between control (*n* = 5) and heat stress (*n* = 5) mice in hippocampal tissues, we identified a reduction in the expression of the *atp2a* gene encoding SERCA, accompanied by a corresponding decrease in its protein level. Consequently, this dysregulation resulted in an excessive accumulation of intracellular calcium ions. Furthermore, HS exposure also activated ERS and apoptosis, as evidenced by the upregulation of p-PERK, p-eIF2α, CHOP, and caspase-3. Consistently, a reduction in postsynaptic density protein 95 (PSD95) and synaptophysin (SYN) expressions indicated modifications in synaptic function. Notably, the impacts on neurons caused by HS were found to be mitigated by CDN1163 treatment both in vivo and in vitro. Additionally, SERCA-mediated ERS-induced apoptosis was attenuated by GSK2606414 treatment via inhibiting PERK-eIF2α-CHOP axis that not only curtailed the level of caspase-3 but also elevated the levels of PSD95 and SYN. These findings highlight the significant impact of heat stress on cognitive impairment, and further elucidate the underlying mechanism involving SERCA/PERK/eIF2α pathway.

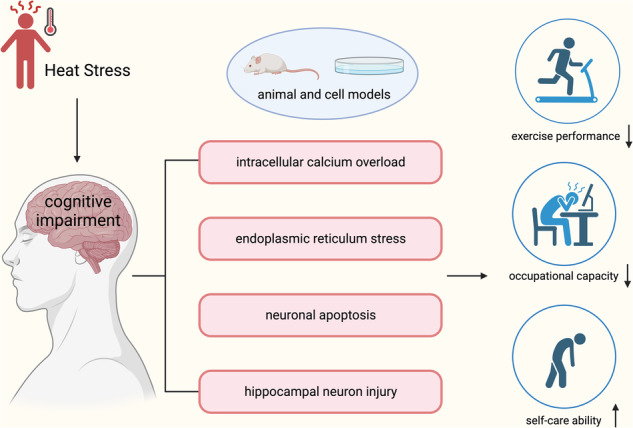

## Highlights

- Heat exposure as an important environmental stressor can profoundly induce cognitive impairment, as presented by learning and memory decline.
- Heat stress initially induces SERCA downregulation and intracellular calcium overload to subsequent ERS-mediated apoptosis and neuronal damage.
- Targeting SERCA/PERK/eIF2α axis might be a promising therapeutic approach to prevent cognitive deficits subsequent to heat stress.

## Introduction

Climate change is driving a worldwide increase in average temperatures and exacerbating the duration, frequency, and intensity of extreme heat events, leading to unprecedented levels of heat exposure [1–3]. Heat stress (HS) is characterized by a cascade of physiological responses that are elicited upon exposure to elevated temperatures. The prolonged duration of this thermal insult disturbs the body’s thermoregulatory mechanisms leading to diverse pathophysiological manifestations including dehydration, tachycardia, anorexia, fatigue, and adversely affected cognitive abilities [4–6]. Increasing pieces of evidence demonstrate that heat stress primarily affects the central nervous system [7, 8], with even brief exposure to hyperthermic conditions potentially resulting in neurological and cognitive dysfunction [9, 10]. However, the precise mechanisms underlying cognitive impairments induced by HS remain inadequately elucidated.

The endoplasmic reticulum plays a crucial role in the intricate network of cellular reticulation and is widely distributed throughout neurons. It serves numerous functions in various cellular processes. When exposed to harmful stimuli or physiological changes, the ER undergoes a process called the unfolded protein response (UPR), which triggers a signaling cascade. This response is initiated to improve protein folding, facilitate quality control mechanisms, and restore ER homeostasis by activating degradative pathways. The three transmembrane sensors of the endoplasmic reticulum, namely inositol-requiring enzyme (IRE1), Protein kinase RNA-like ER kinase (PERK), and activated transcription factor 6 (ATF6), detect misfolded proteins and transmit this signal across the membrane to induce ERS in the cytosol [11]. In cases where the endoplasmic reticulum stress (ERS) reaches an extreme or prolonged state that cannot be resolved, the UPR pathway undergoes a shift from a pro-survival to a proapoptotic state. This shift triggers the activation of PERK, which mediates the phosphorylation of eukaryotic initiation factor 2α (eIF2α). Consequently, there is an upregulation of downstream effectors, including C/EBP-homologous protein (CHOP) [12, 13]. A recent study has reported that exposure to heat induces endoplasmic reticulum (ER) stress, leading to PERK phosphorylation and subsequent apoptosis induction in spermatocytes [14]. Other studies show that ERS is intricately associated with the initiation and progression of neurodegenerative disorders. Specifically, ERS not only results in neuronal loss but also impedes the synthesis of synaptic proteins, thereby impacting cognition and memory function [15–17]. Notably, the impact of disrupted ER homeostasis on cognitive impairment resulting from heat stress has yet to be investigated.

Calcium is predominantly stored within ER, and its equilibrium is tightly linked to ER homeostasis. As expected, the disruption of calcium balance in the endoplasmic reticulum (ER) inevitably leads to the activation of coping mechanisms against ER stress, including the UPR. Minor imbalances in ER calcium levels have been associated with human disorders such as cardiovascular disease and neuropathies [18]. The sarco/endoplasmic reticulum (SR/ER) calcium (Ca^2+^)-ATPase (SERCA) pump is responsible for removing cytoplasmic calcium ions and restoring steady-state levels of calcium ions in the ER. At present, thapsigargin, a SERCA inhibitor, has become a commonly used drug to induce ERS and activate the UPR [19, 20]. More importantly, the dysregulation of Ca^2+^ homeostasis induced by SERCA is implicated in numerous pathological processes characterized by cognitive impairment as the primary clinical manifestation, including Darier’s disease, Alzheimer’s disorder, and cerebral ischemia [21–23]. It is believed that targeting SERCA to restore Ca^2+^ homeostasis and alleviate neural apoptosis caused by ERS holds promise as a potential therapeutic strategy for neurodegenerative diseases. Unfortunately, there is currently no research available to clarify the potential effects of SERCA dysfunction-induced endoplasmic reticulum stress on cognitive decline after HS.

In this study, We present the compelling impact of heat exposure as a prominent environmental stressor on cognitive function, specifically impaired learning and memory abilities. This effect is associated with a downregulation of SERCA, resulting in cytosolic calcium overload, ERS-mediated apoptosis, and subsequent neuron injury to the hippocampus, which can be prevented by activation of SERCA.

## Results

### Heat exposure leads to a decline in cognitive performance and damage to hippocampal neurons

**Fig. 1:**
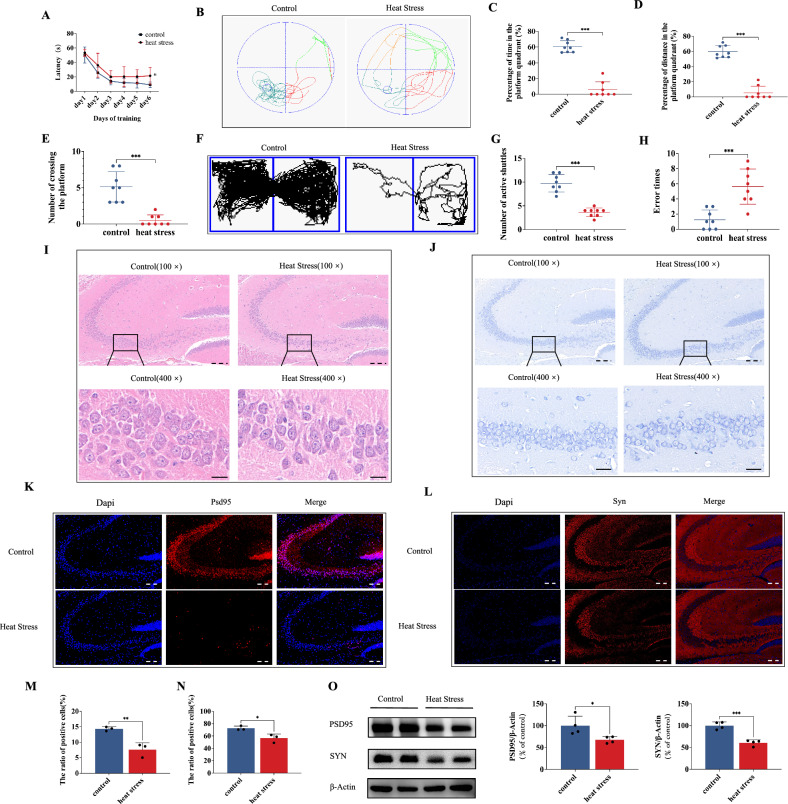
Heat exposure leads to a decline in cognitive performance and damage to hippocampal neurons. **A** Latency in the orientation navigation test. **B** Trajectory map in the spatial probe test. **C**–**E** The percentage of time/ distance of mice swimming in the platform quadrant and the times of crossing of the platform in the spatial probe test. **F** Trajectory map in the shuttle box test. **G** The active shuttle times in the shuttle box test. **H** The error times in the jumping stage test. **I**, **J** HE staining and Nissl staining. Scale bar “--” =20 μm and Scale bar “—” = 100 μm. **K**–**N** Immunofluorescence staining was used to determine the alterations in PSD95 and SYN. Scale bar “--” =20 μm. **O** Representative western blot bands of PSD95 and SYN in the hippocampus of mice. Values are expressed as means ± SD.*p < 0.05, **p < 0.01, ***p < 0.001.

**Fig. 2:**
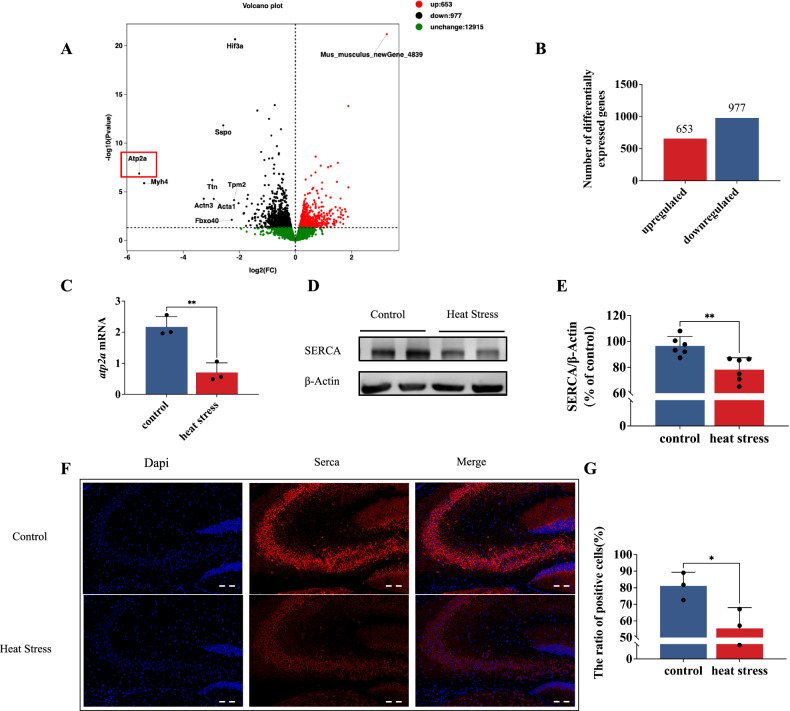
Heat exposure induces SERCA downregulation in hippocampal tissues. **A** Volcano plot shows differential genes. **B** Number of differentially expressed gene between the control and HS mice. **C** *atp2a* mRNA expression of hippocampal tissues (ΔΔct relative quantitative). **D**, **E** Representative western blot bands of SERCA in hippocampal tissues. **F**, **G** Immunofluorescence staining was used to determine the alterations in SERCA. Scale bar “--” =20 μm. Values are expressed as means ± SD.*p < 0.05, **p < 0.01, ***p < 0.001.

To explore the mechanism of cognitive impairment induced by heat stress, we previously established a murine model. Based on this model, we assessed learning and memory ability by conducting the MWM. Despite a decrease in both time and path length required to locate the hidden platform over 6 days of training for all animals, the HS group mice exhibited a significantly prolonged latency compared to the control group (Fig. 1A). Furthermore, the HS group demonstrated a significant reduction in the percentage of time and distance spent swimming within the platform quadrant and the number of times it crossed the target platform during spatial exploration experiments (Fig. 1B–E). Similar results were also obtained in terms of shuttle box examination and jumping stage evaluation (Fig. 1F–H). These phenomena suggest that exposure to HS significantly impairs cognitive function. Then we tested the effects of HS on neuronal morphology and synapse alteration of hippocampal neurons. The hippocampal regions exhibited a consistent and compact arrangement of neurons, as observed through H&E and Nissl staining. The control group showed no significant cell loss, with distinct nuclei present in the neurons. In contrast, neurons in the HS displayed a less organized structure with indistinct cell boundaries, heightened nuclear condensation, and inconspicuous nuclei (Fig. 1I, J). Next, the impact of HS on synaptic protein expression was investigated. The western blot results revealed a marked decrease in the expressions of PSD95 and SYN in the hippocampus following HS exposure, with decreases of 32 and 39%, respectively, compared to the control group (Fig. 2O). These findings were further confirmed by immunofluorescence results (Fig. 2K–N).

### Heat exposure induces SERCA downregulation in hippocampal tissues

To investigate the precise mechanism of HS-induced cognitive damage, we conducted transcriptome sequencing to identify key molecular and signaling pathways. To ensure reliable data for further analysis, we confirmed a parallel group correlation coefficient r greater than 0.962 (see Supplementary Table 1). The volcano plot revealed differential gene expression, identifying a total of 1630 differentially expressed genes (log2 (fold change) ≥ 1 and *P* value < 0.05). Among them, 653 genes were significantly up-regulated while 977 genes were down-regulated, among which the *atp2a* gene encoding SERCA exhibited the most significant downregulation after HS (Fig. 2A, B). We subsequently validated these findings by employing RT-PCR and all the gene expression was normalized to *β-actin* (Fig. 2C), western blot (Fig. 2D, E), and immunofluorescence (Fig. 2F, G) analyses of hippocampal tissues. All the assays consistently revealed a significant reduction in both gene and protein expressions of SERCA following heat exposure.

### SERCA-mediated intracellular calcium overload contributes to endoplasmic reticulum stress and apoptosis following heat stress

**Fig. 3:**
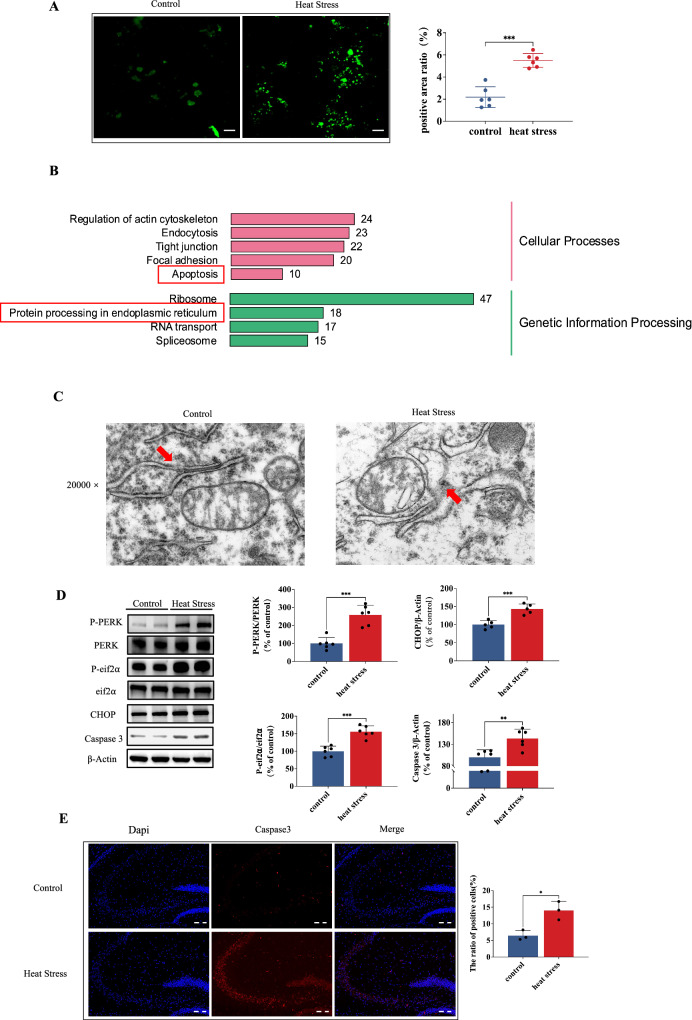
SERCA-mediated intracellular calcium overload contributes to endoplasmic reticulum stress and apoptosis following heat stress. **A** Laser scanning confocal microscope observation and quantification of calcium ions in hippocampus of heat-stressed mice. Scale bar “—” =100 μm. **B** KEGG pathway enrichment analysis for differentially expressed gene. **C** The effect of heat stress on neuronal endoplasmic reticulum was observed under transmission electron microscope; Arrows indicated morphological swelling changes of endoplasmic reticulum. Scale bar = 500 nm. **D** Representative western blot bands of P-PERK, PERK, P-eIF2α, eIF2α, CHOP and Caspase-3 in hippocampal tissues. **E** Immunofluorescence staining was used to determine the alterations in Caspase-3. Scale bar “--” =20 μm. Values are expressed as means ± SD. *p < 0.05, **p < 0.01, ***p < 0.001.

SERCA, located in the ER membrane, pumps excessive Ca^2+^ into the ER to maintain cellular calcium balance. A malfunction of SERCA can lead to cytosolic Ca^2+^ overload. Therefore, we utilized the Fluo-4AM optical probe to detect Ca^2+^ levels in hippocampal tissues and observed a significant increase in intracellular Ca^2+^ levels with HS treatment compared to the control group (Fig. 3A). The literature extensively supports the notion that an imbalance in calcium homeostasis, particularly an excessive accumulation of calcium, can precipitate the initiation of ERS and UPR. Additionally, The protein processing in the ER pathway was enriched by Kyoto Encyclopedia of Genes and Genomes (KEGG) analyses, based on RNA sequencing data (Fig. 3B). As shown in Fig. 3C, the ultrastructure of the endoplasmic reticulum showed abnormal expansion and swelling, mainly manifested by the disappearance of normal folded structures. Furthermore, the protein expressions of p-PERK, p-eIF2α, and CHOP exhibited significant elevations following HS (Fig. 3D). The susceptibility of hippocampal neurons to ERS is noteworthy, as excessive ERS can trigger signaling pathways leading to cell death. The KEGG pathway enrichment analyses suggest that the apoptosis pathway may play a significant role in cognitive damage following HS, as shown in Fig. 3B. Additionally, the protein expression level of caspase-3 was significantly augmented by HS (Fig. 3D), as confirmed by immunofluorescence analysis revealing a marked increase in fluorescence intensity of caspase-3 within the hippocampal tissue of HS-exposed mice (Fig. 3E).

### CDN1163, a SERCA agonist, reverses heat exposure-induced cellular changes in HT22 cells

**Fig. 4:**
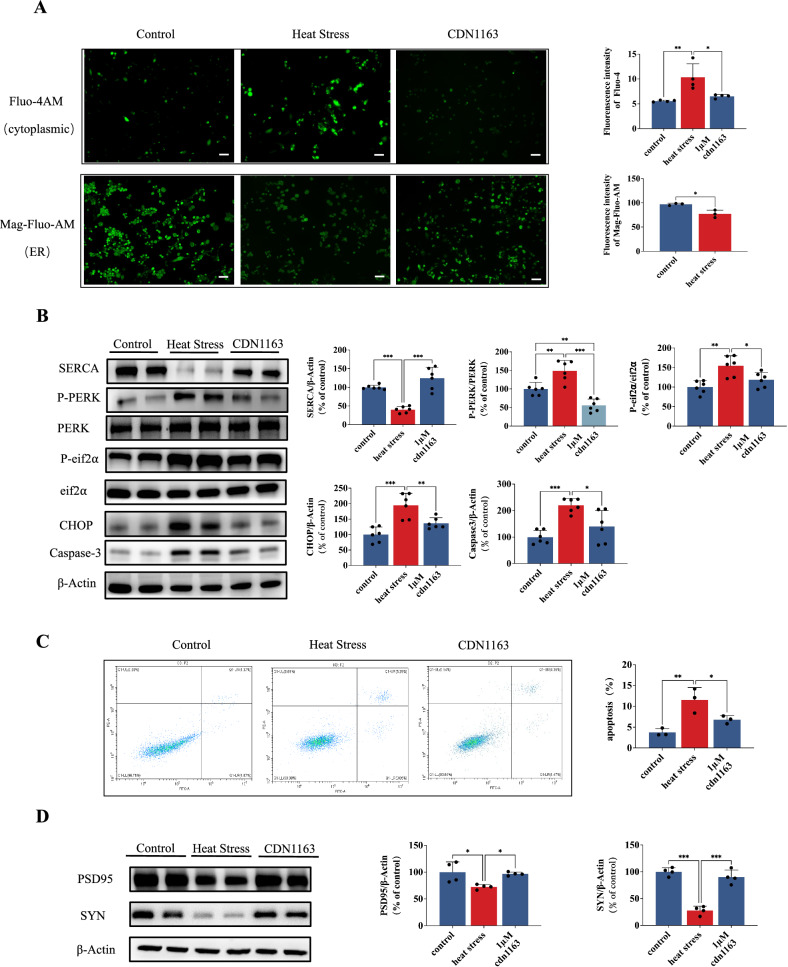
CDN1163, a SERCA agonist, reverses heat exposure-induced cellular changes in HT22 cells. **A** Fluorescence microscope was used to observe and quantitate calcium ions in cytoplasmic and endoplasmic reticulum of HT22 cells. Scale bar “—” =20 μm. **B** Representative western blot bands of SERCA, P-PERK, PERK, P-eIF2α, eIF2α, CHOP and Caspase-3 in HT22. **C** Flow cytometry was used to detect the apoptosis of HT22 cells and quantitative analysis was performed. **D** Representative western blot bands of PSD95 and SYN in HT22. Values are expressed as means ± SD. *p < 0.05, **p < 0.01, *** *p* < 0.001.

To comprehensively investigate the pivotal role of SERCA under heat stress conditions in vitro, HT22 cells were subjected to CDN1163 treatment combined with heat stress to assess the effects on SERCA activation. We observed downregulation of both gene and protein levels of SERCA in response to heat stress, while treatment with CDN1163 significantly mitigated the effects of the heat stress, thereby confirming the activating effects of CDN1163 (Fig. 4B). The treatment of CDN1163 effectively mitigates HS-induced calcium imbalance in HT22 cells, as evidenced by the reduction in cytoplasmic Ca^2+^ levels and the elevation of ER Ca^2+^, as shown in Fig. 4A. Furthermore, the expressions of p-PERK and p-eIF2α proteins were found to be increased in the heat stress group. However, intervention with CDN1163 significantly inhibited their levels, validating that activation of SERCA can effectively attenuate ER stress (Fig. 4B). Additionally, we utilized western blot and flow cytometry techniques to evaluate the levels of caspase-3 and apoptosis mark, respectively, unveiling that CDN1163 effectively counteracted the HS-induced increase in apoptosis (Fig. 4B, C). Similar results were also observed in terms of PSD95 and SYN expressions (Fig. 4D).

### Inhibition of PERK attenuates the apoptosis induced by SERCA downregulation in HT22 cells following heat stress

**Fig. 5:**
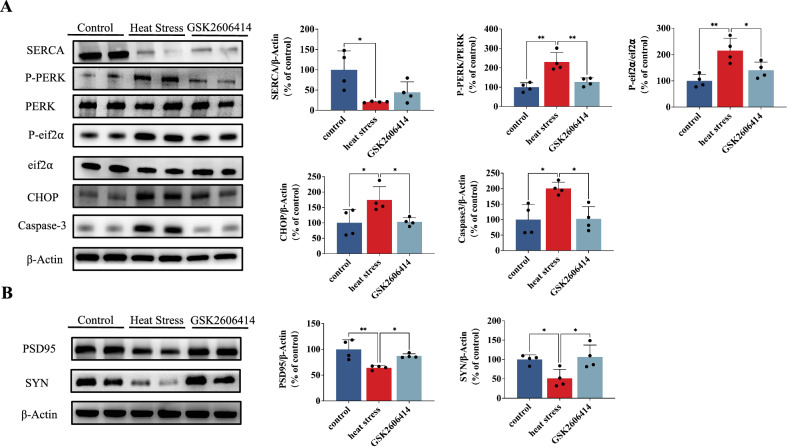
Inhibition of PERK (GSK2606414) attenuates the apoptosis induced by SERCA downregulation in HT22 cells following heat stress. **A** Representative western blot bands of SERCA, P-PERK, PERK, P-eIF2α, eIF2α, CHOP and Caspase-3 in HT22. **B** Representative western blot bands of PSD95 and SYN in HT22. Values are expressed as means ± SD. *p < 0.05, **p < 0.01, ***p < 0.001.

We have demonstrated that inhibition of SERCA expression induces heat stress-induced ER stress and apoptosis, while activation of SERCA effectively mitigates these phenomena. To investigate the role of downregulation of SERCA in apoptosis through the endoplasmic reticulum stress (ERS) pathway, we utilized a PERK inhibitor, GSK2606414, to restore ER function and observe subsequent changes. The inhibitory efficacy of GSK2606414 against PERK was confirmed by the significant reduction in p-PERK protein expression (Fig. 5A). Therefore, we employed GSK2606414 for subsequent investigations. As shown in Fig. 5A, the administration of GSK2606414 did not increase SERCA protein levels. However, it significantly reduced the expressions of p-eIF2α, CHOP, and caspase-3 compared to the heat stress group. Additionally, the PERK inhibitor attenuated the decrease in PSD95 and SYN expressions caused by heat stress (Fig. 5B). Collectively, these findings suggest that SERCA functions as the upstream regulator of PERK, while the PERK/eIF2α/CHOP signaling pathway is involved in apoptosis and modulation of synaptic protein expression mediated by downregulation of SERCA.

### CDN1163 protects cognitive function by reducing endoplasmic reticulum stress and apoptosis in mice exposed to heat

**Fig. 6:**
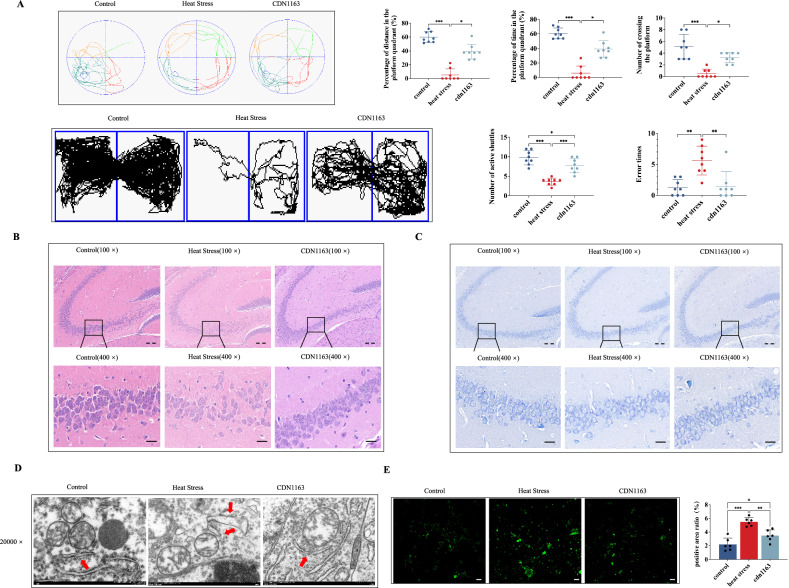
CDN1163 protects cognitive function by reducing damage to hippocampal neurons in mice exposed to heat. **A** Trajectory map in the spatial probe test, the percentage of time/ distance of mice swimming in the platform quadrant and the times of crossing of the platform in the spatial probe test, trajectory map in the shuttle box test, the active shuttle times in the shuttle box test, and the error times in the jumping stage test. **B**, **C** HE staining and Nissl staining. Scale bar “--” =20 μm and Scale bar “—” = 100 μm. **D** The effect of CDN1163 on neuronal endoplasmic reticulum was observed under transmission electron microscope; Arrows indicated morphological swelling changes of endoplasmic reticulum, and the scale was 500 nm. **E** Laser scanning confocal microscope observation and quantification of calcium ions in hippocampus of CDN1163 mice. Scale bar “—” =100μm. Values are expressed as means ± SD. *p < 0.05, **p < 0.01, ***p < 0.001.

**Fig. 7:**
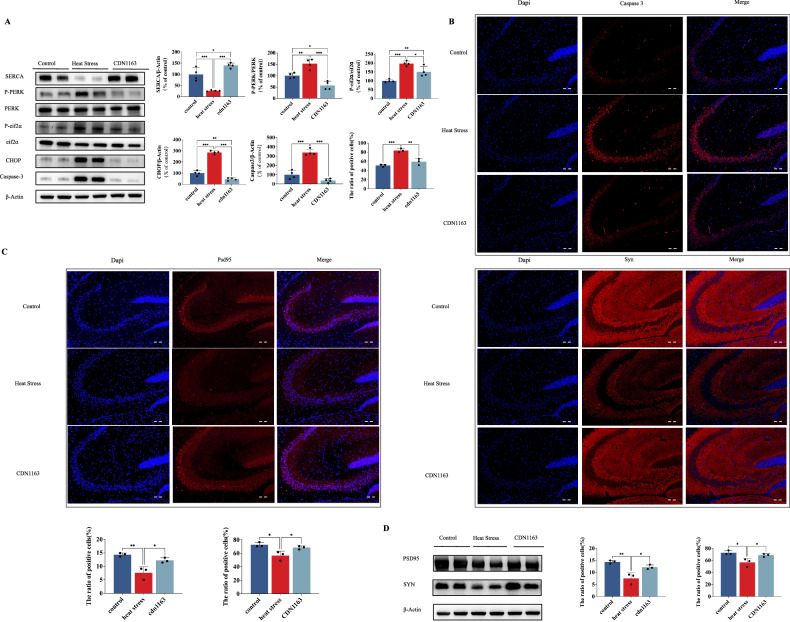
CDN1163 protects cognitive function by reducing endoplasmic reticulum stress and apoptosis in mice exposed to heat. **A** Representative western blot bands of P-PERK, PERK, P-eIF2α, eIF2α, CHOP, and Caspase-3 in hippocampal tissues. **B** Immunofluorescence staining was used to determine the alterations in Caspase-3. Scale bar “--” =20 μm. **C** Immunofluorescence staining was used to determine the alterations in PSD95 and SYN. Scale bar “--” =20 μm. **D** Representative western blot bands of PSD95 and SYN in hippocampal tissues. Values are expressed as means ± SD. *p < 0.05, **p < 0.01, ***p < 0.001.

To further illustrate the function of SERCA obtained above, we used CDN1163 (20 mg/kg) intraperitoneal injections for 7 days to establish SERCA overexpression models for heat exposure test. As demonstrated in Fig. 6F, the expression of SERCA was effectively upregulated by CDN1163 compared to HS. Subsequently, we conducted MWM, shuttle box test, and jumping stage test to evaluate the protective effects of CDN1163 on learning and memory ability after HS. The activation of SERCA expression significantly improved the quadrant time and distance percentage of the platform during the MWM, as well as the number of times it crossed the platform, the number of active shuttles in the shuttle box test, and the error times in the jumping stage test (Fig. 6A). Notably, administration of CDN1163 reversed the morphological changes in hippocampal tissue and compromised ER ultrastructure (Fig. 6B–D). The calcium image depicted that CDN1163 predominantly decreased the cytosolic Ca^2+^ level and restored Ca^2+^ homeostasis compared to the HS group (Fig. 6E). In addition, we tested ERS and apoptosis marker changes after CDN1163 treatment in heat stress. The administration of CDN1163 effectively induced reductions in the protein expressions of p-PERK, p-p-eIF2α, CHOP, and caspase-3 following HS (Fig. 7A). Furthermore, there was a concomitant decrease in the intensity of caspase-3 immunofluorescence induced by HS (Fig. 7B). Finally, we investigated whether CDN1163 influenced the modulation of PSD95 and SYN expressions, which were found to be downregulated in the HS group relative to the control group. Fortunately, both PSD95 and SYN western blot as well as immunofluorescence displayed significant elevations in response to CDN1163 stimulation (Fig. 7C, D).

## Discussion

**Fig. 8:**
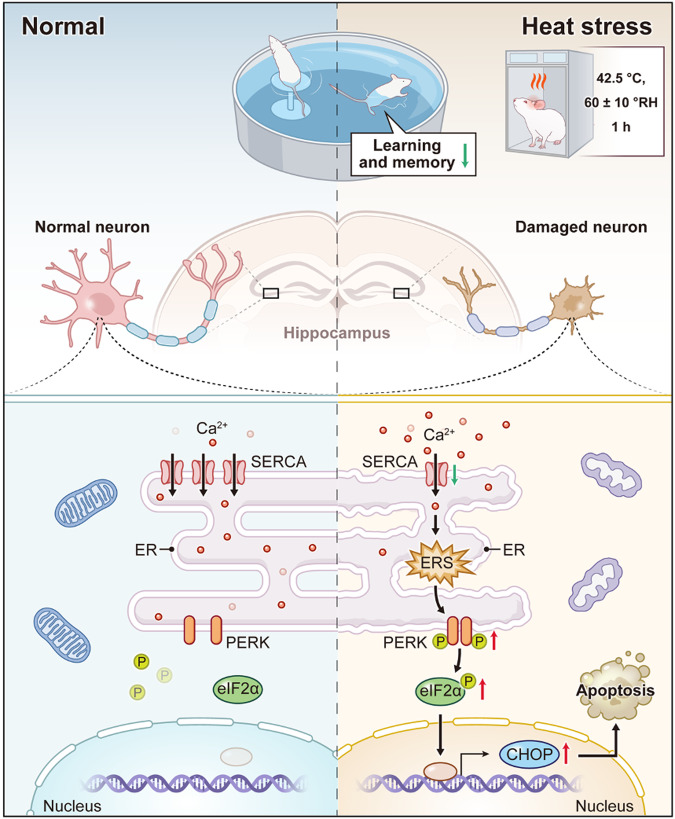
The schematic diagram shows the possible mechanism of cognitive decline caused by heat exposure. Heat stress can cause cognitive impairment by damaging neurons and synapses, and with the down-regulation of SERCA, p-PERK/p-eIF2α/CHOP signaling pathway is activated, leading to apoptosis.

Exposure to heat stress elicits a plethora of physiological, histological, and molecular modifications in the brain [24–26]. Despite extensive investigations into the physiological responses triggered by heat stress, its impact on cognitive function and the precise underlying mechanism remains elusive. In the current study, utilizing an in vivo and in vitro heat stress model, we have unveiled the crucial role of SERCA in mediating cognitive impairment induced by heat stress. Our findings demonstrate that heat stress leads to downregulation of SERCA, resulting in calcium imbalance. This subsequently triggers ESR and ERS-mediated apoptosis, ultimately leading to the loss of functional neurons and neuronal synapses. Importantly, these phenomena can be reversed through the activation of SERCA. Based on our results, we proposed a potential therapeutic target for mitigating cognitive impairment following heat stress (Fig. 8).

According to the murine heat stress model with learning and memory impairment we established previously [27], heat significantly induces declines in learning and memory abilities as assessed by the orientation navigation, the spatial probe, the shuttle box, and the jumping stage tests. Additionally, it is widely believed that changes in synaptic connectivity play a crucial role in the processes underlying learning and memory. The postsynaptic proteins PSD95 and SYN play crucial roles in maintaining synaptic stability and plasticity in neurons. Excitatory neurons modulate their expression levels to regulate synaptic function, and a significant decrease in their expression is strongly linked to cognitive damage in neurodegenerative disorders [28, 29]. Our findings from the histological staining, western blot, and immunofluorescence revealed that heat stress led to neuronal loss and downregulation of postsynaptic protein markers. Thus, we hypothesized that the heat-induced cognitive impairment may be attributed, at least in part, to neuronal and synaptic loss. However, the specific mode of cell death and underlying mechanism remained unaddressed. The bioinformatics analysis conducted by our team revealed the potential involvement of SERCA and apoptosis in the development of neuronal loss and cognitive decline following exposure to heat stress. We observed that the coding gene SERCA, *atp2a*, exhibited the most significant differential expression among all 1630 genes analyzed. Additionally, it was found to be associated with apoptosis as a predominant form of cell death.

SERCA, a well-established integral protein of the ER, plays an important role in regulating intracellular Ca^2+^ signaling by effectively maintaining low cytosolic Ca^2+^ levels through ATP hydrolysis-mediated facilitation of free Ca^2+^ ion flux into the ER lumen [22, 23, 30, 31]. However, its role in the central nervous system remains elusive, particularly under heat exposure conditions. In the present study, we initially identified its differential expression through transcriptional screening and subsequently investigated its response to heat stress in both in vivo and in vitro settings using multiple techniques including RT-PCR, western blot, and immunofluorescence. Our findings consistently demonstrated a significant reduction in SERCA expression upon exposure to heat stress. Moreover, we observed an increase in cytosolic Ca^2+^ levels and a decrease in ER levels through Ca^2+^ imaging tests, indicating disruption of Ca^2+^ homeostasis and cytosolic Ca^2+^ overload. The term ‘Ca^2+^ homeostasis’ represents a relatively stable process, upheld by dynamic Ca^2+^ fluxes occurring between the ER Ca^2+^ reservoir, the cytosol, and the extracellular space [32]. To elucidate the primary cause of cytosolic Ca^2+^ overload resulting from SERCA downregulation following heat stress, we employed CDN1163, a small allosteric activator of SERCA, to restore SERCA functionality and observe changes in intracellular Ca^2+^ level. The activation of SERCA mediated by CDN1163 has been extensively documented; Kang et al. reported the enhancement of SERCA activity in vivo and stimulation of Ca^2+^ uptake in vitro by CDN1163 [33]. Importantly, the administration of CDN1163 affected learning and memory in the MWM test and ameliorated symptomatology associated with Alzheimer’s disease (AD) [34] and Parkinson’s disease (PD) [35] in rodent models. Our results demonstrated that treatment with CDN1163 effectively induced SERCA expression while preserving its effects under heat stress conditions. Additionally, intracellular Ca^2+^ levels tended to be balanced, as evidenced by a significant reduction in cytosolic Ca^2+^. Most importantly, there was a notable improvement in learning and memory ability, attenuation of apoptosis, and mitigation of neuronal damage, when compared to HS. In summary, the present study proposes a crucial pathological function of SERCA in facilitating HS-induced activation of apoptosis and cognitive impairment.

The UPR serves as a cellular survival mechanism triggered by the presence of misfolded or unfolded proteins within the ER. While the UPR aims to restore cellular homeostasis, persistent and excessive activation of ER stress pathways, such as heat stress, can contribute to pathological UPR and ER-dependent proapoptotic signaling [15, 36]. Specifically, a recent study has reported that exposure to HS induces ERS and activates the p‐eIF2α/CHOP pathway, leading to compromised integrity of the epithelial barrier through induction of apoptosis in intestinal epithelial cells [37]. Another study has demonstrated the significance of ERS markers, namely ATF6 and PERK, as crucial mediators in heat-induced apoptosis in spermatocytes [14]. However, to the best of our knowledge, the involvement of ERS and UPR in HS-induced apoptosis and cognitive impairment remains elusive, particularly regarding the role of SERCA-mediated pathways. Recently, inhibition of SERCA has emerged as a prevalent approach to induce ERS and the UPR [38]. Furthermore, a study demonstrated that pharmacologically targeting SERCA dysfunction, CDN1163 can effectively restore ER Ca^2+^ levels, mitigate ER stress, and attenuate ER stress-induced cell death in the liver by inhibiting PERK/eIF2α/CHOP [33]. In our current study, we observed a reduction in the heat-induced elevations of phosphorylation of PERK (p-PERK) and its downstream target elF2α (p-elF2α), as well as a decrease in the expression of the pro-apoptotic transcription factor CHOP upon CDN1163 administration both in vivo and in vitro. In addition, we used the PERK inhibitor GSK2606414 to investigate the role of SERCA dysfunction-induced PERK/elF2α/CHOP signaling activation in mediating heat stress-induced apoptosis in HT22 cells. Studies have demonstrated that GSK2606414 effectively inhibits the PERK-eIF2α axis, thereby attenuating neuronal apoptosis induced by endoplasmic reticulum stress, improving neuropathological damage, memory, and motor function impairments, and preventing neurodegeneration in Parkinson’s disease model [39]. In this study, we initially observed no significant change in SERCA expression when compared to the HS group, suggesting that PERK may function downstream of SERCA and that heat exposure might first induce alterations in SERCA levels, subsequently leading to endoplasmic reticulum stress (ERS). The expressions of p-elF2α, CHOP, and caspase-3 were significantly reduced, along with the synaptic proteins PSD95 and SYN. Overall, these findings suggest the involvement of the PERK/elF2α/CHOP signaling pathway in apoptosis and neuronal damage following the downregulation of SERCA after HS.

## Conclusion

In conclusion, this study provides compelling evidence that heat exposure is a significant environmental stressor with profound implications for cognitive function, particularly in terms of learning and memory abilities. Moreover, we have elucidated a novel mechanism whereby the downregulation of SERCA leads to the activation of the p-PERK/p-elF2α/CHOP signaling pathway, resulting in subsequent apoptosis. Consequently, this cascade promotes neuronal injury and contributes to the development of cognitive impairment following heat stress. Targeting the inhibition of SERCA represents a promising strategy for the mechanism that exacerbates injury and provides a novel therapeutic avenue for addressing post-HS cognitive decline.

## Materials and methods

### Animals and cells

Animal studies were performed under the approval of the Institutional Animal Care and Use Committee of Naval Medical University, following the guidelines of “Guide for the Care and Use of Laboratory Animals”. C57BL/6 mice, sample size estimation, and compliance with ethical regulations were described in our previous publication and were obtained, housed, and treated according to the protocol outlined in our previous study [27]. Briefly, mice were divided into three groups of eight mice each: ① control group; ② heat stress group: 42.5 ± 0.5 °C, 60 ± 10% relative humidity, 60 min per day for 7 days; ③ CDN1163 (CDN1163, a small molecule SERCA activator) group: rats were exposed to heat as the heat stress group every day, and CDN1163 20 mg/kg was injected intraperitoneally 1 h before heating. No blinding was established.

HT22 mouse hippocampal neurons were obtained from Shanghai Fuheng Technology Co., Ltd (Shanghai, China) and cultured following the provided guidelines. The neurons were cultured in high glucose DMEM supplemented with 10% FBS and maintained at 37 °C with 5% CO_2_. The HT22 hippocampal neurons were cultured in 6-well plates with a cell density of 2 × 10^5^ cells per well and allowed to incubate for 12 h. Then, the plates were divided into the following groups: ① control group: cultured at 37 °C for 48 h; ② HS group: cultured at 41 °C for 48 h; ③ CDN1163 group (CDN1163): cultured for 24 h at 41 °C, then treated with 1 µM CDN1163 and cultured for another 24 h at 41 °C; ④ GSK2606414 group (GSK2606414): after incubation for 24 h at 41 °C, 1 µM GSK2606414 was given and incubated for another 24 h at 41 °C. All plates were subjected to fluid exchange or drug administration 24 h after the start of treatment.

### Reagents

The compounds CDN1163 (CAS No.: 892711-75-0) and GSK2606414 (CAS No.: 1337531-36-8) were obtained from MedChemExpress Co., Ltd.

### Behavioral experiments

The procedures of the Morris water maze (MWM), shuttle box test, and jumping stage test are conducted according to the previous methodology [27]. In summary, a 6-day experiment on positioning navigation was conducted, where mice were randomly placed in water from four different entrance directions each day. The time spent on the positioning platform was recorded. The subsequent stage involves space exploration testing. In this stage, the platform is eliminated, and the mice are introduced into the pool starting from the furthest quadrant for 1 min. The testing for the shuttle box system includes 3 days of training and 1 day of testing. Following 5 min of free exploration, a 15-s red light and buzzer will be activated, followed by 5 s of electrical stimulation. Once the mouse enters a safe area, the stimulation will cease, and this process will be considered a cycle. The training phase will last for 20 cycles, while the testing phase will last for 30 cycles. The jumping phase will last for 2 days, with the initial day designated as the training session and the subsequent day allocated for testing purposes. During the initial 3-min adaptation period, mice will be placed in a box. Subsequently, the insulation platform will be activated by the stainless steel grille located at the bottom of the box, prompting the mouse to swiftly move onto it to prevent any potential electric shock. The training session is scheduled for a duration of 5 followed by a subsequent test phase that will take place 24 h after completing the training.

### Samples collection

After the completion of behavioral testing, the mice were given anesthesia. Samples of blood were obtained from the ocular region, and the obtained serum samples were preserved at −80 °C in a refrigerator for future use, following centrifugation at 8000 × *g* for 10 min. Following that, some mice underwent cardiac perfusion with 4% paraformaldehyde, and collected the hippocampal tissues for HE staining and immunofluorescence. The remaining mice received cardiac perfusion solely with normal saline. While a portion of the hippocampus tissue was immediately utilized for calcium fluorescence probe research and RNA sequencing, another portion was stored in electron microscopy stationary liquid for transmission electron microscopy. The remaining hippocampus tissue was preserved in a freezer at −80 °C for further tests.

### HE staining, nissl staining, and immunofluorescence

For HE staining, the sections, approximately 4 μm thick, underwent the following staining procedure: hematoxylin staining for 5 min, rinsing with water until a blue color appeared, eosin staining for 3 min, dehydration, sealing, air-drying, and observation under an optical microscope.

The slides were air-dried at room temperature for at least 60 min before Nissl staining. The sections were rinsed twice in 10 mM PBS for 5 min each ddH_2_O for 1 min. After that, they were immersed in Nissl stain solution for 20 min. Following staining, two more rounds of rinsing with ddH_2_O were performed, each lasting 5 min. Subsequently, the slides underwent a series of ethanol and xylene treatments: first in 90% ethanol for 3 min, then in 95% ethanol for another set of 3 min; subsequently undergoing two for durations of 3 each time; finally being subjected to three changes with pure (100%) xylene for durations of 3 min per change. Seal with neutral gum and observe under the microscope.

For immunofluorescence, the section thickness was about 4 μm. The sections were positioned within an immune response enhancement solution and subjected to microwave treatment for 15 min. After cooling and washing, the sections were blocked with BSA for 30 min. The SERCA (1:100, ab150435, Abcam, UK) and PSD95 (1:100, GB11277-100, Servicebio, China) antibodies were applied overnight at 4 °C, followed by incubation with the respective secondary antibodies for 50 min. Subsequently, DAPI staining was performed for 10 min. After washing, The slides were sealed with tablets that prevent fluorescence quenching, and images were captured using fluorescence microscopy (LICOR, USA).

### Transmission electron microscopy (TEM)

The mouse hippocampus tissue was freshly dissected and sectioned into approximately 1 m^3^ cubes. Subsequently, the tissue cubes were treated with a 2.5% glutaraldehyde fixative solution and kept at a temperature of 4 °C for storage. Following proper washing, the samples underwent a dehydration process and were then embedded using a combination of acetone and 812 embedding agents. Polymerization was achieved by placing the samples in an oven set at 60 °C for 48 h. The resulting blocks were then sliced into ultra-thin sections with a thickness ranging from 60–80 nm. Finally, these sections were subjected to staining and observed through transmission electron microscopy.

### Ca^2+^ imaging of hippocampal tissue and HT22 cells

The freshly prepared hippocampal tissues were transferred to glass slides treated with poly-lysine after being sheared and placed in a PBS solution. To enhance the binding of Fluo-4 AM, a concentration of 2 µM, and ensure even distribution, 20% Pluronic F-127 (Beyotime, China) was carefully added drop by drop onto the hippocampal tissues using a micro sampler. The treated cover glass was then applied to press the slide, ensuring the uniform spreading of the hippocampal tissue with consistent force. After an incubation period of 30 min at 37 °C, the samples were gently rinsed twice with a PBS solution and subsequently supplemented with one drop of PBS solution. The fluorescence intensity was recorded using a laser confocal microscope (Nikon Eclipse Ti, Japan). When excited at 488 nm, the Fluo-4 AM bound to calcium ions emitted green fluorescence. Specific fluorescent probes, Fluo-4 AM (Beyotime, China), and Mag-Fluo-AM (Genmed Scientific S Inc., USA) were used to measure cytoplasmic and endoplasmic reticulum Ca2+ concentrations, respectively. HT22 cells were harvested from the old culture medium and washed three times with PBS. Fluo-4 AM (2 µM) was added to completely cover the cells, followed by incubation at 37 °C for 30 min. After three subsequent washes with PBS, the cells were further incubated for an additional 30 min to allow complete conversion of Fluo-4 AM into Fluo-4 in the cells. Cytoplasmic calcium ions were then observed using a fluorescence microscope. The cells were trypsinized, and the suspended cells were counted in the culture medium. A small amount of culture medium was dropwise added into a 12-well plate, and glass slides were placed in the wells. The cells were added to each well and allowed to incubate for 12 h for grouping. After the grouping treatment, the supernatant was discarded, and 500 µl of washing solution was used to rinse the cells. A staining working solution was then prepared by mixing 30 µl of the mediator solution and 1.5 µl of the staining solution with 300 µl of the washing solution. The staining working solution was carefully added and incubated at room temperature in the dark for 60 min. Following the incubation period, the working solution was discarded, and the calcium ions in the endoplasmic reticulum were immediately observed under a fluorescence microscope using a laser wavelength of 490 nm and an emission wavelength of 525 nm.

### Flow cytometry

The evaluation of apoptosis was conducted by utilizing the Annexin V-FITC/PI Apoptosis Detection Kit (Yeasen, China). HT22 cells were detached by trypsinization without EDTA, and centrifuged 5 min at 4 °C. Discarding the supernatant, and washing cells twice with PBS, each time undergoing a 300 × *g* centrifugation at 4 °C for 5 min. After removing the PBS, a 100 µl solution of 1× binding buffer was used to resuspend the cells. An aliquot of Annexin V-FITC (5 µl) and PI (10 µl) holding solution was introduced, gently agitated, and subjected to a light-free incubation period lasting 15 min. Then, 1× buffer (400 µl) was added, and thoroughly mixed, and the samples were ready for analysis using a suitable machine.

### RNA sequencing and bioinformatics

The Trizol reagent (Life Technologies, USA) was utilized for the extraction of total RNA, followed by purification using the RNeasy mini kit (Qiagen, Germany). The concentration and purity of the RNA were then determined using the NanoDrop 2000 (Thermo Fisher Scientific, Wilmington, DE). To evaluate RNA integrity, the RNA Nano 6000 test kit from the Agilent Bioanalyzer 2100 System (Agilent Technologies, CA, USA) was employed. Subsequently, a sequencing library was generated using the Herefs Ultima DUAL-MODE mRNA Library Prepkit for Illumina (Yeasen Biotechnology (Shanghai) Co., Ltd), following the manufacturer’s instructions. RNA-seq was conducted on the Illumina NovaSeq 6000 platform (Baimaikeyun, China).

### RT- PCR

The RNA extraction utilized test kits and followed the steps outlined in the protocol (RNAiso Plus, Takara, Japan). The extracted RNA was then diluted with RNase-free ddH2O. Following the extraction, the reverse transcription process was performed following the guidelines provided by the manufacturer using a reverse transcription kit from Takara, Japan. The *atp2a gene* sequence: (F) TGGAACAACCCGGTAAAGAGT; (R) CACCAGGGGCATAATGAGCAG; *β-Actin:* (F) GGCTGTATTCCCCTCCATCG; (R)CCAGTTGGTAACAATGCCATGT.

### Western blot

To analyze the protein expressions obtained from HT22 cells and hippocampus, SDS-PAGE gels with varying concentrations of 10%, 12.5%, or 15% were used. Subsequently, the proteins were moved onto an NC membrane and exposed to a blocking solution for 15 min (Epizyme, Shanghai, China). After blocking, the membrane was incubated overnight at 4 °C with primary antibodies, including SERCA (1:20,000, ab150435, Abcam, UK), PERK (phospho T980) (1:1000, AP328, Beyotime, China), PERK (1:1000, ab229912, Abcam, UK), Phospho-eIF2α (1:1000, AF1237, Beyotime, China), eIF2α (1:2500, ab169528, Abcam, UK), Caspase-3 (1:2000, ab184787, Abcam, UK), PSD95 (1:2500, AF1237, Beyotime, China), and SYN (1:20,000, ab32127, Abcam, UK). The following day, the secondary antibody (1:100,000, LICOR, USA) labeled with an infrared fluorescent dye was applied and incubated for 1 h. The visualization process was accomplished by utilizing a dual-color infrared fluorescence imaging system from LICOR, USA. The intensity of the band was standardized by normalizing it to the intensity of the β-actin protein band.

### Statistical analysis

Data were presented as mean ± standard deviation (SD), and the GraphPad software was used for all statistical analyses. The Shapiro-Wilk test was used to check the normality and homogeneity of variance of all data. The orientation navigation test was analyzed by two-way ANOVA. The remaining data correspond to the following methods. For two-group comparisons, Student *t*-tests were used to determine differences between groups for normally distributed data. For multiple group comparisons, *p* values were analyzed using one-way ANOVA or Kruskal–Wallis test. All tests were two-sided, and *p* < 0.05 was considered statistically significant.

## Supplementary information

supplemental table 1

supplemental material

## Data Availability

All original data are available from the corresponding author upon request.
