# Correction: Heat stress induces calcium dyshomeostasis to subsequent cognitive impairment through ERS-mediated apoptosis via SERCA/PERK/eIF2α pathway

**DOI:** 10.1038/s41420-024-02124-x

**Published:** 2024-09-04

**Authors:** Hongxia Li, Wenlan Pan, Chenqi Li, Mengyu Cai, Wenjing Shi, Zifu Ren, Hongtao Lu, Qicheng Zhou, Hui Shen

**Affiliations:** 1 Department of Nutrition and Food Hygiene, Faculty of Naval Medicine, Naval Medical University, Shanghai, 200433 China; 2 Department of Clinical Nutrition, Shanghai Ninth People’s Hospital, Shanghai JiaoTong University School of Medicine, Shanghai, 201999 China; 3 Department of Nutrition, The Third Affiliated Hospital of Naval Medical University, Shanghai, 200438 China

**Keywords:** Disorders of consciousness, Hippocampus

Correction to: *Cell Death Discovery* (2024) 10.1038/s41420-024-02047-7, published online 11 June 2024

In this article the contribution section read:

These authors contributed equally: Hongxia Li, Wenlan Pan, Chenqi Li. These authors jointly supervised this work: Hongtao Lu, Qicheng Zhou, Hui Shen

It should read:

These authors contributed equally: Hongxia Li, Wenlan Pan, Chenqi Li.

These authors jointly supervised this work: Hongtao Lu, Qicheng Zhou, Hui Shen.

The original article has been corrected.

