# Supplementary material for: Heat stress induces calcium dyshomeostasis to subsequent cognitive impairment through ERS-mediated apoptosis via SERCA/PERK/eIF2α pathway
Source: Cell Death Discov. 2024 Jun 11;10:280. doi: 10.1038/s41420-024-02047-7 (PMC11167007; doi:10.1038/s41420-024-02047-7)
Supplement: Supplementary file 1 — supplemental table 1 [file 41420_2024_2047_MOESM1_ESM.docx]

Table 1 Heat map of expression quantity correlation between samples

|  | C1 | C2 | C3 | C4 | C5 | HS1 | HS2 | HS3 | HS4 | HS5 |
| --- | --- | --- | --- | --- | --- | --- | --- | --- | --- | --- |
| C1 | 1 | 0.9802 | 0.983 | 0.9615 | 0.9648 | 0.9926 | 0.9748 | 0.9682 | 0.9709 | 0.9693 |
| C2 | 0.9802 | 1 | 0.9985 | 0.9905 | 0.9908 | 0.9914 | 0.9915 | 0.9858 | 0.9935 | 0.9926 |
| C3 | 0.983 | 0.9985 | 1 | 0.9892 | 0.9897 | 0.9916 | 0.9894 | 0.9855 | 0.9928 | 0.9908 |
| C4 | 0.9615 | 0.9905 | 0.9892 | 1 | 0.9983 | 0.971 | 0.977 | 0.9685 | 0.9895 | 0.9902 |
| C5 | 0.9648 | 0.9908 | 0.9897 | 0.9983 | 1 | 0.9735 | 0.975 | 0.974 | 0.9932 | 0.9936 |
| HS1 | 0.9926 | 0.9914 | 0.9916 | 0.971 | 0.9735 | 1 | 0.9895 | 0.9862 | 0.9844 | 0.9824 |
| HS2 | 0.9748 | 0.9915 | 0.9894 | 0.977 | 0.975 | 0.9895 | 1 | 0.9795 | 0.9816 | 0.9793 |
| HS3 | 0.9682 | 0.9858 | 0.9855 | 0.9685 | 0.974 | 0.9862 | 0.9795 | 1 | 0.9921 | 0.9891 |
| HS4 | 0.9709 | 0.9935 | 0.9928 | 0.9895 | 0.9932 | 0.9844 | 0.9816 | 0.9921 | 1 | 0.9978 |
| HS5 | 0.9693 | 0.9926 | 0.9908 | 0.9902 | 0.9936 | 0.9824 | 0.9793 | 0.9891 | 0.9978 | 1 |

Table.1 Pearson's correlation coefficient R was used as an evaluation index of biological repetitive correlation. Values are expressed as means ± SD.* *p* < 0.05，* * *p* < 0.01，* * * *p* < 0.001.
