# Supplementary material for: Heat stress induces calcium dyshomeostasis to subsequent cognitive impairment through ERS-mediated apoptosis via SERCA/PERK/eIF2α pathway
Source: Cell Death Discov. 2024 Jun 11;10:280. doi: 10.1038/s41420-024-02047-7 (PMC11167007; doi:10.1038/s41420-024-02047-7)
Supplement: Supplementary file 2 — supplemental material [file 41420_2024_2047_MOESM2_ESM.docx]

Figure 1O:

PSD95

SYN

β-actin

Figure 2D：

SERCA

β-actin

Figure 3D：

P-PERK

PERK

P-eif2⍺

eif2⍺

CHOP

β-actin

Caspase-3

β-actin

Figure 4B

SERCA

P-PERK

PERK

P-eif2⍺

eif2⍺

CHOP

Caspase-3

β-actin

Figure 4D：

PSD95

SYN

β-actin

Figure 5A：

SERCA

P-PERK

PERK

P-eif2⍺

eif2⍺

CHOP

Caspase-3

β-actin

Figure 5B：

PSD95

SYN

β-actin

Figure 7A：

SERCA

P-PERK

PERK

P-eif2⍺

eif2⍺

CHOP

Caspase-3

β-actin

Figure 7D：

PSD95

SYN

β-actin
